# A novel method for efficient delivery of stem cells to the ischemic brain

**DOI:** 10.1186/scrt327

**Published:** 2013-09-27

**Authors:** Ling Guo, Jianfeng Ge, Shan Wang, Ying Zhou, Xiaoxiao Wang, Yaojiong Wu

**Affiliations:** 1 The Shenzhen Key Laboratory of Health Sciences and Technology, Graduate School at Shenzhen, Tsinghua University, Shenzhen, China; 2 School of Life Sciences, Tsinghua University, Beijing, China

**Keywords:** Stroke, Acute cerebral infarction

## Abstract

**Introduction:**

Rat middle cerebral artery occlusion (MCAO) model is the most commonly used animal model in ischemic stroke studies. In the model, to increase the amount of stem cells or drugs to enter the brain after delivery into the internal carotid artery (ICA), the pterygopalatine artery (PPA) is occluded. However, PPA occlusion is a technically demanding procedure which often causes complications.

**Methods:**

In this study, we developed an ICA injection needle to facilitate easy and efficient delivery of stem cells to the ischemic brain through the ICA without the need of PPA occlusion. We injected methylene blue and fluorescence dye DiI-labeled human mesenchymal stem cells (DiI-hMSCs) into the ICA in rats with the ICA injection needle (without PPA ligation) or the conventional micro-injection needle (with PPA ligation) and assessed their distributions.

**Results:**

When methylene blue was injected, evident blue stains were found in the brain of the injection side particularly the middle cerebral artery (MCA)-supplied areas but not in the PPA supplied areas. Similarly, when DiI-hMSCs were injected, the cells largely appeared in the MCA-supplied tissues, which were similar in quantity compared to conventional micro-injection needle injection with PPA occlusion. Moreover, hMSCs injected with the ICA needle or the micro-injection needle similarly improved the functional recovery of the infarcted brain.

**Conclusions:**

Our results indicate that the ICA injection needle is easy to use and efficient in delivering cells to the ischemic brain tissue in rat MCAO model.

## Introduction

Stroke is a leading cause of death and disability. Stem cells have demonstrated profound potential in the repair and regeneration of the injured brain tissue [1]. The therapeutic effect of stem cells to an injured organ is often associated with the number of stem cells engrafted. Several routes have been used to deliver stem cells for brain injuries in previous studies, such as intravenous injection, intra-arterial injection and intra-cerebral administration. The intra-cerebral approach involves complicated and invasive procedures while the intravascular routes are safer and more applicable. The intravascular paradigm relies on the ability of stem cells to migrate toward injured tissues in a targeted manner, mediated by endogenous chemoattractant and adhesion molecules. Although intravenous administration is the least invasive and the most convenient route, the majority of intravenously delivered stem cells are trapped in the lungs and other organs and do not reach the target organ [2-4]. Intra-arterial administration bypasses the filter of the lungs and facilitates a larger number of cells to reach the target organ. Indeed, it has been shown that intra-arterial injection leads to a higher and sustained cell presence at the ischemic site compared to intravenous administration [5,6].

Rat ischemic stroke models have been used extensively to evaluate the benefit of stem cells and to study the underlying mechanisms. Since ischemic stroke in patients is mostly caused by the occlusion of the middle cerebral artery (MCA), intraluminal suture MCA occlusion (MCAO) has become the most commonly used model in the investigation of stroke in rodents [7]. The reversible MCAO model has been used widely due to its clinical relevance in that it mimics recanalization of the thrombosed MCA vessel in a clinical situation. Stem cells or drugs can be injected into the internal carotid artery (ICA) for targeted delivery to the reperfused brain tissue. Because the pterygopalatine artery (PPA) is a branch of the ICA, blood in the ICA will shunt into the PPA supplied region. Therefore, most researchers occluded the PPA when they performed intra-arterial injection [8]. However, ligation of the PAA is a difficult procedure because of the position of this artery [9]. Moreover, occlusion of the PPA for a long time may cause thrombosis and lead to severe consequences, such as embolism in MCAO reperfusion. Therefore, it is desirable to develop a method to temporarily occlude the PPA when stem cells or drugs are delivered into the ICA.

In this study, we developed an ICA injection needle according to the anatomic features of the ICA and the PPA. The needle has a closed end to occlude the PPA and a lateral opening to deliver stem cells into the ICA. We tested the needle by injecting methylene blue and fluorescence dye DiI-labeled human mesenchymal stem cells (hMSCs) into the ICA in rats and assessing their distribution. Our results showed that no blue stain was found in the PPA-supplied regions and similar amounts of DiI-hMSCs appeared in the MCA-supplied brain tissues compared to the PPA occlusion technique.

## Methods

### ICA injection needle

**Figure 1:**
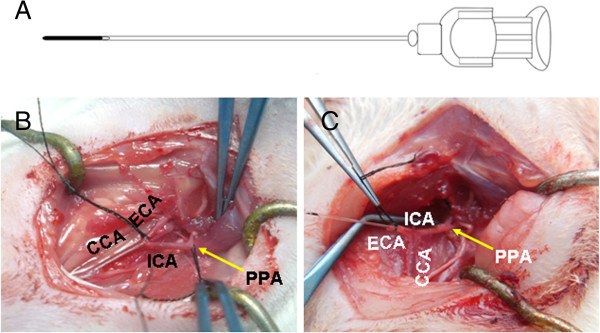
**The ICA injection needle and anatomy of carotid arteries. (A)** A schematic diagram of the ICA needle. **(B)** Anatomy of the common carotid artery (CCA), external carotid artery (ECA) and internal carotid artery (ICA). **(C)** After cutting down the distal end of the ECA, pull the ECA to make it straight to the ICA. The ICA injection needle was inserted from the distal end of the ECA and advanced into the ICA and the pterygopalatine artery (PPA).

The ICA injection needle is similar to a regular syringe needle, but has several modifications. The needle is 3.0 to 4.0 cm long, with external diameters of 0.3 to 0.5 mm. The tip of the needle is blunt and closed ended. At 5 mm from the tip, there is a 27G opening, which serves as the exit of injected materials through the needle (Figure 1A). The front segment of the ICA needle is solid, and the length of the rest of the needle is similar to that of the conventional needle, so the volume retained in the needle is almost equal to that in the conventional micro-injection needle. The size of the needle is selected depending on the body weight of the rat. For rats weighing 250 to approximately 270 grams, a 3.5-cm long and 0.4-mm external diameter needle is appropriate.

The ICA injection needle was designed based on the anatomic structure of the ICA and the PPA in rats (Figure 1B-C). In rats, the common carotid artery divides into the external carotid artery and the ICA at the thyroid gland level. At about 5 mm from the bifurcation, the ICA has a branch called the PPA (Figure 1B-C). The trunk and the branch of the ICA and the PPA form a ‘Y’ shaped structure. When the ICA injection needle is inserted into the ICA, this special structure facilitates the closed ended needle to be easily advanced into the PPA to temporarily block the blood flow. The external diameter of the needle is designed to fit the internal diameter of the PPA. The lateral opening of the needle is 5 mm away from the tip. This distance allows a deep insertion of the needle into the PPA lumen to sufficiently occlude the blood flow and ensure the opening is toward the ICA.

**Figure 2:**
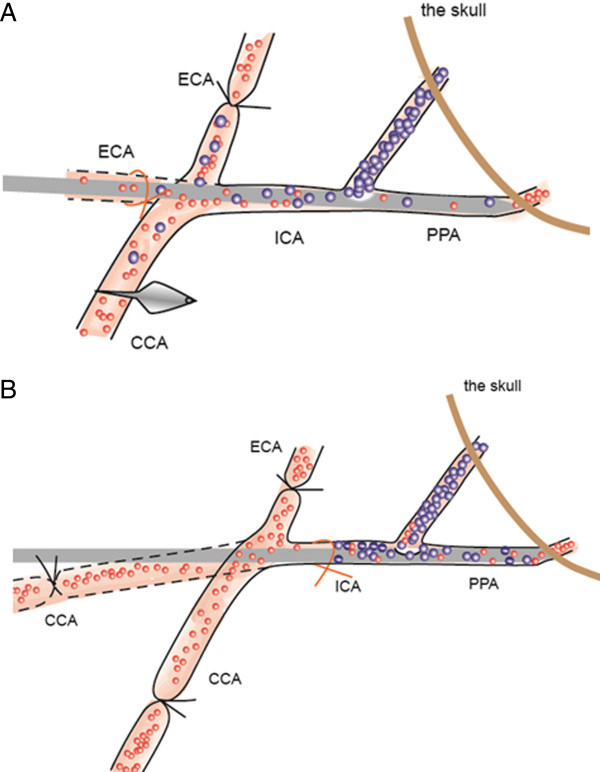
**Two insertion routes of the ICA injection needle are shown. (A)** Insertion of the ICA injection needle into the internal carotid artery (ICA) via the external carotid artery (ECA) stump. Pull the ECA to make it straight to the ICA. Insert the needle into the ECA and advance the needle into the ICA and the pterygopalatine artery (PPA). **(B)** Insertion of the ICA injection needle into the ICA via the common carotid artery (CCA). Pull the CCA to make it straight to the ICA. Insert the needle into the CCA and advance the needle into the ICA and the PPA.

The ICA injection needle was inserted into the ICA from the external carotid artery. First, the external carotid artery was ligated with a nylon suture and nicked at a 45-degree angle. Then the external carotid artery was pulled to make it straight to the ICA (Figure 2A). The needle was inserted into the external carotid artery, then advanced into the ICA and further into the PPA and stopped when an obvious resistance was felt. The ligature was tightened at the external carotid artery stump until hemostasis was achieved. Solutions (cells or saline) were infused slowly through the needle. When completed, the needle was withdrawn and the external carotid artery stump was ligated. Alternatively, the ICA needle could be inserted into the ICA from the common carotid artery if the common carotid artery is pulled to make it straight to the ICA (Figure 2B).

### Animals

Female Sprague–Dawley rats (250 to 270 g, purchased from the Laboratory Animal Centre, Guangdong Province, China) were used in the study. Rats were maintained in a temperature controlled environment (20 ± 1°C) with access to food and water throughout the experiment. All procedures were performed with the approval of the Animal Ethics Committee of Tsinghua University.

### Middle cerebral artery occlusion and reperfusion in rats

After anesthesia with 10% chloral hydrate (400 mg/kg, intraperitoneal (i.p.) injection, Sigma-Aldrich), rats were subjected to transient focal ischemia for 120 minutes by the intraluminal suture technique as described previously [10] with medications. Briefly, the common carotid artery, external carotid artery and ICA on the left side were carefully exposed via a midline cervical incision. The external carotid artery was ligated with a 4–0 silk suture (10 mm from the bifurcation), and the common carotid artery was clamped with a microsurgical clip. A silicon rubber-coated round-tip nylon surgical thread was inserted into the ICA (approximately 18 mm from the bifurcation) from the external carotid artery stump to occlude the origin of the MCA. A silk suture around the external carotid artery was tightened to prevent bleeding from the puncture site, and the clip was removed. After 120 minutes of MCAO, reperfusion was performed by withdrawing the suture.

### Methylene blue injection

Rats were divided into three groups and each group had four animals. In group 1, the PPA was ligated with a 6–0 silk suture. A total of 200 μl methylene blue (20 mg/2 ml, Jiangsu Jumpcan Pharmaceutical Group Co., Ltd., Taizhou City, China) was injected with a 30G micro-injection needle (Hamilton) into the common carotid artery with preserved flow in the common carotid artery and ICA. In group 2, 200 μl methylene blue was injected with the ICA injection needle into the internal carotid artery. The needle was inserted into the ICA from the external carotid artery stump and advanced towards the head of the rat, so the closed-ended needle tip was inserted into the PPA to occlude the PPA, thus preventing the methylene blue from entering the PPA, and the methylene blue was delivered into the ICA via the lateral opening of the needle thus facilitating its entry into the brain. In group 3, the ICA was ligated. A total of 200 μl methylene blue was injected into the PPA with a micro-injection needle. Rats were scarified immediately after injection of methylene blue. The skin of the head was removed to observe the distribution of methylene blue in facial tissues. Then the brain was exposed to observe the distribution of methylene blue. Finally, coronal sections through the brain were cut into seven equally spaced blocks (2 mm each, including a block of cerebellum) to examine methylene blue distribution inside the brain.

### Labeling and transplantation of human mesenchymal stem cells

Human MSCs were isolated and characterized in our laboratory as previously described [11]. Briefly, the research proposal and the procedure of placenta collection were first approved by the Ethics Committee of Shenzhen Futian Hospital. Term (38 to 40 weeks gestation) placentas, which were otherwise discarded, were collected from hospitalized healthy donors with written informed consent. Placenta tissue was cut into small pieces and enzymatically digested into a single cell suspension, from which MSCs adherent to uncoated plastic tissue culture dishes were isolated. The cells were cultivated in a growth medium comprising (Dulbecco’s) modified Eagle’s medium ((D)MEM, Sigma-Aldrich), 10% fetal bovine serum (Gibco) 100 IU per ml penicillin and 100 μg/ml streptomycin, and passage 4 to 5 cells were used for xenotransplantation to rats. To label hMSCs, monolayer hMSCs were incubated with lipophilic fluorochrome 1,1′-dioctadecyl-3,3,3′,3′-tetramethylindocarbocyanine perchlorate (DiI, Sigma-Aldrich) in serum- and phenol-free (D)MEM at 37°C for 45 minutes as previously described [12,13]. Cells were then incubated in the growth medium at 37°C for one hour, washed with phosphate-buffered saline (PBS) and harvested for transplantation. Two hours after MCAO, 1 million DiI-hMSCs in 100 μl PBS were injected into the ICA in two minutes using three methods: with an ICA needle without PPA ligation; with a micro-injection needle with PPA ligation (the conventional method); or with a micro-injection needle without PPA occlusion.

### Generation of single cell suspensions

Immediately after cell transplantation, the left hemisphere of the brain of the rats was removed carefully and the cerebral tissues were cut into six equally spaced (2 mm) coronal blocks. Fresh tissues of the third block were weighed, ground in a cell strainer and washed with a digestion buffer (0.1% trypsin-ethylenediaminetetraacetic acid (EDTA)) to generate a single cell suspension. DiI-hMSCs were visualized under a fluorescence microscope and the number of hMSCs per 10^4^ cells was counted. Thirty microscopic fields were randomly selected (10 fields/group).

### Histological analysis

The left hemisphere of the brain was removed carefully on day 7 after cell transplantation and the cerebral tissues were cut into six equally spaced (2 mm) coronal blocks. The fourth block was fixed in 4% paraformaldehyde (PFA), embedded in optimal cutting temperature compound (OCT) and cut into 10-μm-thick sections, which included the MCA perfused territory. Tissue sections were visualized under a fluorescence microscope for the presence of DiI-hMSCs and representative fields were photographed. For quantification of DiI-hMSCs in the ischemic boundary zone, cells positive for DiI per microscopic field (×200) were counted, and 10 fields per tissue section were randomly selected. Three tissue sections per sample were examined (n = 4).

### Neurological severity scores

Rats were subjected to a modified Neurological Severity Scores (mNSS) test at 0, 1, 3, 5, 7, 9, 11 and 14 days of MCAO as described previously [14]. mNSS is a composite of motor, sensory, reflex and balance tests. In the severity scores of injury, one point was awarded for the inability to perform the test or for the lack of a tested reflex; thus, the higher the score, the more severe was the injury.

### Statistical analysis

All data were expressed as mean ± standard deviation (SD). Results were analyzed using one-way or repeated measures analysis of variance (ANOVA) with independent variables of treatment groups and days of testing, followed by the Bonferroni post-hoc test for multiple comparisons between treatment groups at each time point. A probability (*P*) value <0.05 was considered significant.

## Results

### Characterization of the ICA injection needle

The ICA injection needle was easily inserted into the ICA from either the external carotid artery or the common carotid artery, and then smoothly advanced into the PPA. In this study, we chose to insert the needle from the external carotid artery to preserve the blood flow in the common carotid artery and in the ICA during stem cell transplantation. As the end of the needle is round and smooth, the procedure did not cause any complications, such as blood vessel rupture and bleeding.

### Distribution of methylene blue

**Figure 3:**
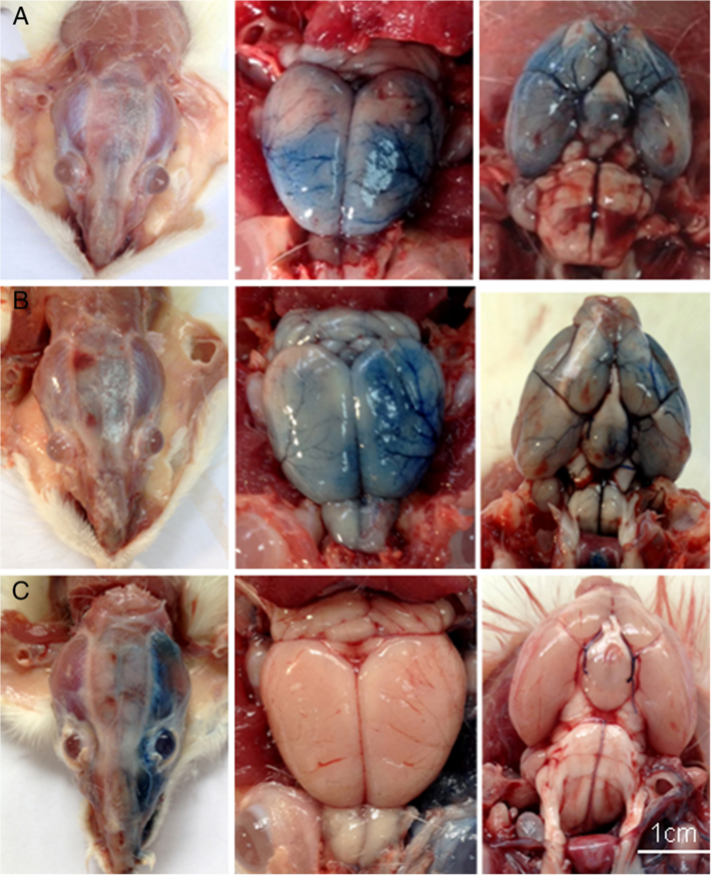
**Distribution of methylene blue in three injection routes. (A)** Injection of methylene blue into the internal carotid artery with a micro-injection needle after ligation of the pterygopalatine artery (PPA). **(B)** Injection of methylene blue into the internal carotid artery with the ICA injection needle without ligation of the PPA. **(C)** Injection of methylene blue into the PPA with a micro-injection needle.

To verify whether PPA was effectively occluded by the closed end of the ICA injection needle, methylene blue was injected through the needle. Blue stained tissues were largely localized to the MCA supplied regions of the brain [See Additional file 1: Figure S1, n = 4], and no blue stains were found in PPA supplied areas including tissues around the eyes, nose and mouth (Figure 3B, n = 4). The dye distribution pattern was similar to that in animals treated with ligated PPA and the conventional micro-injection needle (Figure 3A, n = 4). Conversely, when methylene blue was injected into the intact PPA, blue stains were found in the left side of the face including areas around the eyes, nose and mouth, but no blue stains were found in the MCA supplied regions of the brain (Figure 3C and Additional file 1: Figure S1).

### Distribution of hMSCs

**Figure 4:**
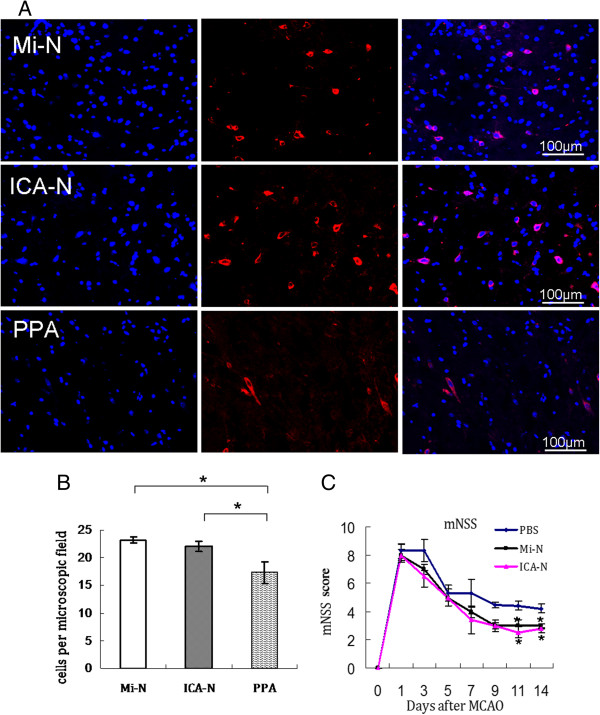
**MSCs in the brain.** Seven days after injection of DiI-hMSCs using three methods in rats with MCAO, tissue sections of the brain were examined under a florescence microscope for the presence of DiI-hMSCs (red). **(A)** Representative images are shown. Mi-N, DiI-hMSCs delivered with a micro-injection needle plus PPA ligation; ICA-N, DiI-hMSCs delivered with an ICA injection needle; C, PPA, DiI-hMSCs delivered with a micro-injection needle without PPA ligation. Nuclei were stained with Hoechst (blue). **(B)** Quantitation of DiI-hMSCs in tissue sections as described in the ‘Methods’ (n = 4, *P <0.05). **(C)** mNSS scores of rats before and after receiving different treatments at times as indicated. hMSCs were injected one day post MCAO before mNSS evaluation. PBS, injection of equal volume of PBS with a micro-injection needle plus PPA ligation. *P <0.05. DiI, 1, 1′-dioctadecyl-3, 3, 3′, 3′-tetramethylindocarbocyanine perchlorate; DiI-hMSCs, DiI-labeled human mesenchymal stem cells; ICA, internal carotid artery; MCAO, middle cerebral artery occlusion; mNSS, modified neurology severity score; PPA, pterygopalatine artery.

To further examine whether the ICA injection needle could efficiently deliver stem cells to the damaged brain without PPA ligation, we compared the number of DiI-hMSCs in tissue sections of the MCA-supplied region of the brain in rats with MCAO seven days after cell injection. Similar amounts of DiI-hMSCs were found in the brain of rats injected with the conventional micro-needle plus PPA ligation or with the ICA needle without PPA ligation, but many fewer DiI-hMSCs were found in the brains of rats injected with a conventional micro-needle without PPA ligation (Figure 4A and B, *P* <0.05). To further quantify the number of the DiI-hMSCs in the brain, equal amounts of brain tissues around the infarct (immediately after cell transplantation) were digested into single cell suspensions, and the number of DiI-positive cells was counted under a fluorescence microscope [see Additional file 2: Figure S2, n = 4]. The results are consistent with the findings in histology (Figure 4A and B).

### Neurological outcomes

The severity of neurological defects was evaluated in rats with MCAO after transplantation of hMSCs using different methods. Prior to MCAO, mNSS scores indicated that there was no neurological defect in any of the rats. One day after MCAO and before MSC transplantation, no significant differences were found in mNSS score among the groups (Figure 4C, *P* >0.05, n = 5). However, at 11 and 14 days post MCAO, rats that had received hMSCs via the conventional micro-needle plus PPA ligation or the ICA needle exhibited significant improvement in neurological function as indicated by the mNSS compared to rats that received only the PBS injection (Figure 4C, *P* <0.05, n = 5). There were no significant differences in mNSS between rats injected with the conventional micro-needle plus PPA ligation and rats injected with the ICA needle (Figure 4C, *P*> 0.05, n = 5).

## Discussion

Rat MCAO models have been the most commonly used model in stroke studies. To achieve maximum distribution of stem cells or drugs to the ischemic cerebral tissue after delivery into the ICA, the PPA is occluded in the model, although its occlusion is currently considered unnecessary for the establishment of the MCAO model. Due to technical difficulties in performing PPA occlusion and subsequent complications, the application of the MCAO model in research has been greatly limited. In this study, we developed an ICA injection needle which facilitated easy and efficient delivery of stem cells to the MCAO brain via the ICA without the need of PPA occlusion.

According to methods for filament insertion in the MCAO model [10,15], we tested two routes for the insertion of the ICA injection needle. One was from the external carotid artery stump, and the other was from the common carotid artery. From both routes, the ICA injection needle was easily advanced into the PPA. Of them, insertion from the external carotid artery stump is preferred, because the blood flow in both the common carotid and the ICA can be preserved during the entire stem cell transplantation process, and only the external carotid artery remains ligated after surgery. Recent studies indicate that preservation of the blood flow in the common carotid artery and the ICA causes minimal changes in cerebral blood flow during intra-ICA injection and does not cause thromboembolic complications, thereby greatly minimizing surgical influences on the animals [8,15,16].

MSCs are multipotent stem cells capable of differentiating into mesoderm- and nonmesoderm-derived tissues [13,17-19]. They have been identified in a variety of tissues and are likely to participate in the maintenance of stem cell niches and tissue homeostasis [20,21]. Transplantation of MSCs has been shown to enhance the repair and regeneration of various tissues including those affected by strokes [1,22]. Moreover, MSCs have low immunogenicity and show immune regulatory properties [23]. For these reasons, MSCs are emerging as an extremely promising therapeutic agent for a variety of diseases. In this study, we easily injected hMSCs into the ICA in rats with MCAO and reperfusion using the ICA injection needle which temporarily blocked the blood flow to the PPA, thus facilitating maximum entry of the stem cells into the damaged brain. Indeed, we found similar amounts of DiI-hMSCs in the brain of rats receiving DiI-hMSCs injected with the ICA injection needle without PPA ligation which were associated with similar improvements in neurological function compared to rats receiving DiI-hMSCs injected with a micro-injection needle plus PPA ligation. On the same principle, many other cell types, such as neural stem cells, and drugs can be delivered with this needle thereby allowing targeted distribution of them to the damaged brain. Moreover, as the ICA injection needle does not block blood flow in the ICA, it can be used to deliver cells or drugs into the cerebral vasculature without causing cerebral ischemia.

## Conclusions

In this study, we developed an ICA injection needle according to the anatomic features of the ICA and the PPA. The needle has a closed end to occlude the PPA and a lateral opening to deliver stem cells into the ICA. Our data indicate that the needle facilitated easy and efficient delivery of stem cells into the ICA and further to the MCA-supplied area of the brain without the need of PPA ligation, thus avoiding the technical difficulties and complications seen with PPA ligation.

## Abbreviations

(D)MEM: (Dulbecco’s) modified Eagle’s medium; CCA: Common carotid artery; DiI: 1, 1′-dioctadecyl-3, 3, 3′, 3′-tetramethylindocarbocyanine perchlorate; DiI-hMSCs: DiI-labeled human mesenchymal stem cells; ECA: External carotid artery; ICA: Internal carotid artery; MCA: Middle cerebral artery; MCAO: Middle cerebral artery occlusion; mNSS: Modified neurological Severity Scores; MSCs: Mesenchymal stem cells; PBS: Phosphate-buffered saline; PPA: Pterygopalatine artery.

## Competing interests

The authors declare they have no competing interests.

## Authors’ contributions

LG conceived and designed the study, prepared the MCAO model and injections, provided animal care and performed data acquisition, analysis and manuscript writing and revision. JFG provided animal care and performed data acquisition. SW performed the isolation and expansion of MSCs. YZ and XXW performed sample preparation, data entry and manuscript writing. The study was conducted in the laboratory of YJW and he was responsible for supervision of the study and manuscript writing and revision. All authors read and approved the final manuscript.

## Supplementary Material

Additional file 1: Figure S1 The distribution of methylene blue in coronal sections of the brain. (A) Injection of methylene blue into the ICA with a micro-injection needle after ligation of the PPA. (B) Injection of methylene blue into the ICA with an ICA injection needle without PPA ligation. (C) Injection of methylene blue into the PPA with a micro-injection needle. Click here for file

Additional file 2: Figure S2 hMSCs distribution. 10^6^ DiI-hMSCs were injected into the left ICA with a micro-injection needle plus PPA ligation (Mi-N), the ICA injection needle without PPA ligation (ICA-N) or a micro-injection needle without PPA ligation (PPA) in rats with left MCAO. Immediately after cell transplantation, a block of the cerebral tissue in the left MCA-supplied territory per rat was harvested and digested into a single cell suspension, which was subjected to analysis under a fluorescence microscope for the presence of DiI-hMSCs (red) in each field. (A) A representative microscopic field from each group is shown. Nuclei were stained with Hoechst (blue). (B) Quantitation of DiI-hMSCs in the single cell suspensions of a given amount of tissue (n = 4). Click here for file
