# Supplementary figures and images for: A novel method for efficient delivery of stem cells to the ischemic brain
Source: Stem Cell Res Ther. 2013 Sep 27;4(5):116. doi: 10.1186/scrt327 (PMC3854714; doi:10.1186/scrt327)

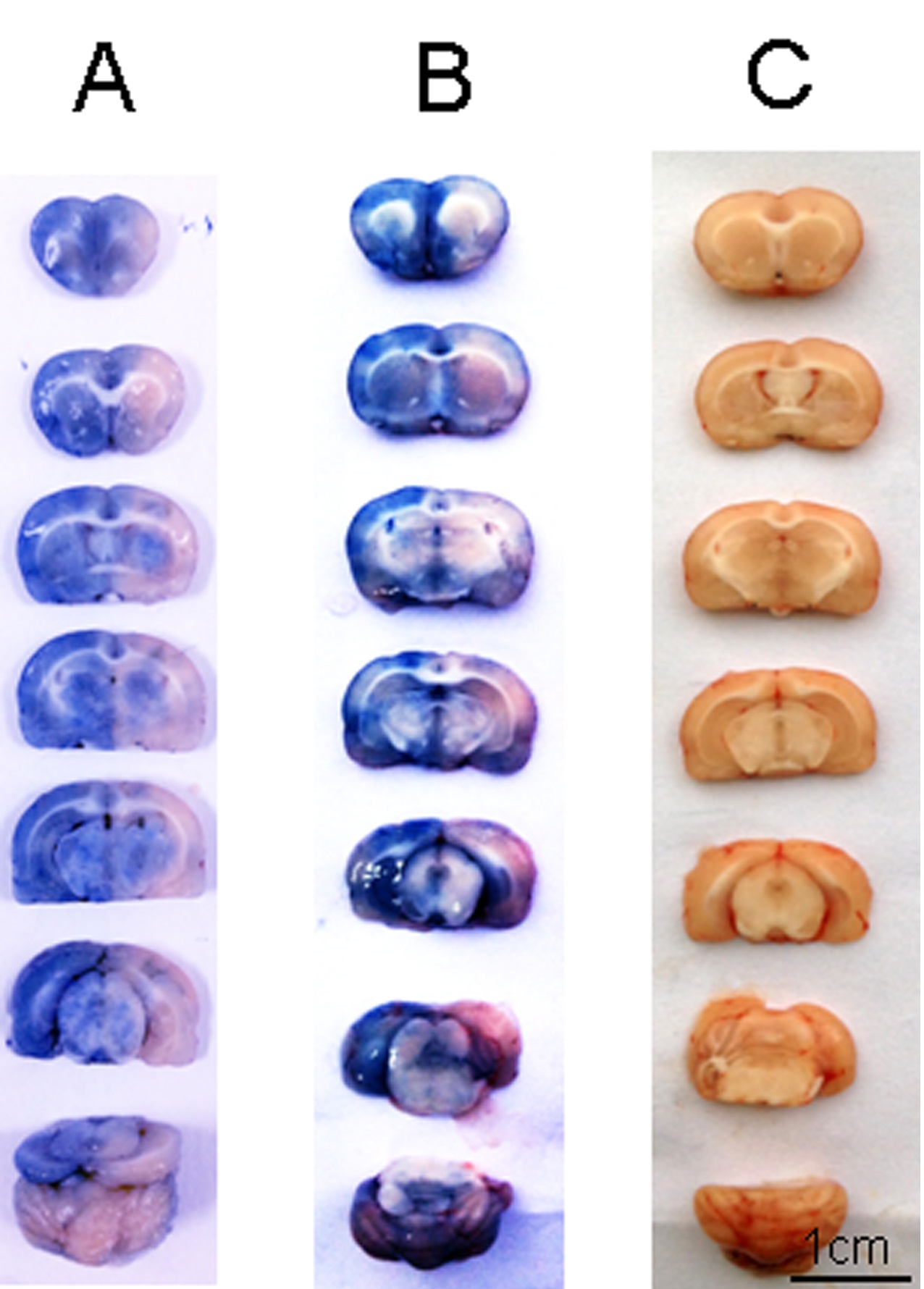

Supplement: Additional file 1: Figure S1 — The distribution of methylene blue in coronal sections of the brain. (A) Injection of methylene blue into the ICA with a micro-injection needle after ligation of the PPA. (B) Injection of methylene blue into the ICA with an ICA injection needle without PPA ligation. (C) Injection of methylene blue into the PPA with a micro-injection needle. [file scrt327-S1.jpeg]

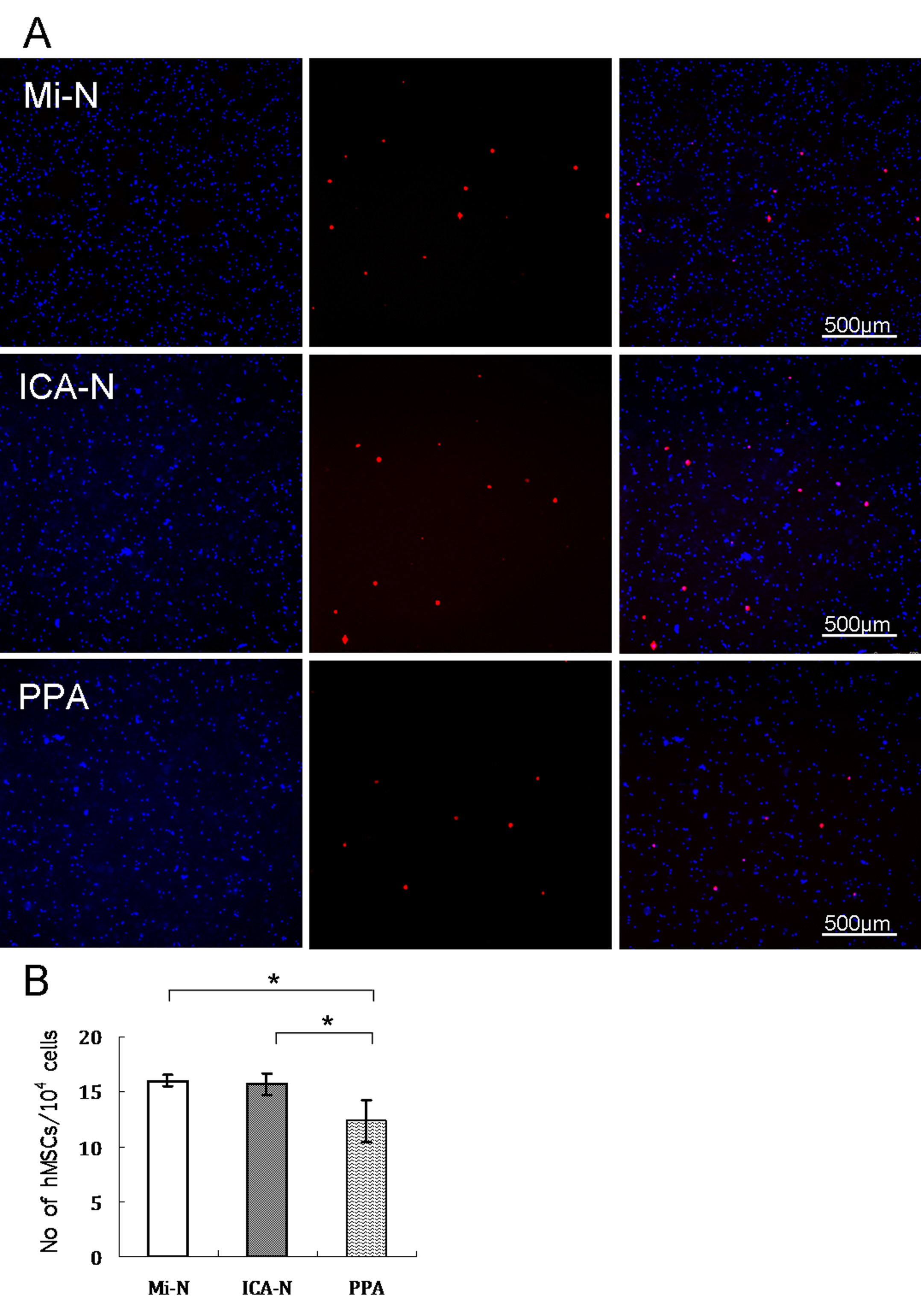

Supplement: Additional file 2: Figure S2 — hMSCs distribution. 106 DiI-hMSCs were injected into the left ICA with a micro-injection needle plus PPA ligation (Mi-N), the ICA injection needle without PPA ligation (ICA-N) or a micro-injection needle without PPA ligation (PPA) in rats with left MCAO. Immediately after cell transplantation, a block of the cerebral tissue in the left MCA-supplied territory per rat was harvested and digested into a single cell suspension, which was subjected to analysis under a fluorescence microscope for the presence of DiI-hMSCs (red) in each field. (A) A representative microscopic field from each group is shown. Nuclei were stained with Hoechst (blue). (B) Quantitation of DiI-hMSCs in the single cell suspensions of a given amount of tissue (n = 4). [file scrt327-S2.jpeg]
